# PUBLIC HEALTH RESPONSES TO DEMENTIA IN RURAL AND TRIBAL AREAS: BOLD AND BEYOND

**DOI:** 10.1093/geroni/igae098.1224

**Published:** 2024-12-31

**Authors:** Katie Spears, Erin Bouldin, Lisa McGuire

**Affiliations:** Chair; Co-Chair; Discussant

## Abstract

Rural and tribal communities have unique characteristics specific to their geographical location and cultures that can influence how disease prevention and health promotion programs are delivered in these areas. Public health officials may need to adapt traditional strategies to improve uptake, effectiveness, and reach in rural and tribal settings. CDC’s Healthy Brain Initiative (HBI) Road Map Series identifies priority actions public health agencies can take to promote brain health and support caregivers. The Road Map Series includes one designed for local and state agencies and another for Indian Country. CDC’s Building Our Largest Dementia (BOLD) Infrastructure for Alzheimer’s Public Health Program awardees carry out Road Map actions to develop and strengthen the public health infrastructure. This session will illustrate how public health entities have developed dementia resources within rural and tribal areas in their jurisdictions. Four states – Maine, Mississippi, Utah, and Washington – and a tribal board – the Northwest Portland Area Indian Health Board – will be presenters. They will characterize their population, identify the dementia programs and services they offer, describe challenges faced, and discuss the impact of rurality and tribes on their approach to service delivery and outreach. Presenters will discuss resources and collaborative approaches used to ensure success. Together, these presentations will highlight public health strategies that have been effective at supporting brain health in all communities across the lifespan.

